# Numerical Homogenization of Orthotropic Functionally Graded Periodic Cellular Materials: Method Development and Implementation

**DOI:** 10.3390/ma17246080

**Published:** 2024-12-12

**Authors:** Behnam Shahbazian, Victor Bautista Katsalukha, Mirmilad Mirsayar

**Affiliations:** Department of Aerospace, Physics, and Space Sciences, Florida Institute of Technology, Melbourne, FL 32901, USA; bshahbazian2021@my.fit.edu (B.S.); vbautista2016@my.fit.edu (V.B.K.)

**Keywords:** periodic functionally graded cellular materials, 2D numerical homogenization, MATLAB code, elasticity tensor, orthotropic materials, isotropic materials

## Abstract

This study advances the state of the art by computing the macroscopic elastic properties of 2D periodic functionally graded microcellular materials, incorporating both isotropic and orthotropic solid phases, as seen in additively manufactured components. This is achieved through numerical homogenization and several novel MATLAB implementations (known in this study as *Cellular_Solid*, *Homogenize_test*, *homogenize_ortho*, and *Homogenize_test_ortho_principal*). The developed codes in the current work treat each cell as a material point, compute the corresponding cell elasticity tensor using numerical homogenization, and assign it to that specific point. This is conducted based on the principle of scale separation, which is a fundamental concept in homogenization theory. Then, by deriving a fit function that maps the entire material domain, the homogenized material properties are predicted at any desired point. It is shown that this method is very capable of capturing the effects of orthotropy during the solid phase of the material and that it effectively accounts for the influence of void geometry on the macroscopic anisotropies, since the obtained elasticity tensor has different $E_{1}$ and $E_{2}$ values. Also, it is revealed that the complexity of the void patterns and the intensity of the void size changes from one cell to another can significantly affect the overall error in terms of the predicted material properties. As the stochasticity in the void sizes increases, the error also tends to increase, since it becomes more challenging to interpolate the data accurately. Therefore, utilizing advanced computational techniques, such as more sophisticated fitting methods like the Fourier series, and implementing machine learning algorithms can significantly improve the overall accuracy of the results. Furthermore, the developed codes can easily be extended to accommodate the homogenization of composite materials incorporating multiple orthotropic phases. This implementation is limited to periodic void distributions and currently supports circular, rectangular, square, and hexagonal void shapes.

## 1. Introduction

The term “cellular structures” is quite descriptive, denoting a medium consisting of a void and solid material (i.e., the matrix), where each void is encased by a solid framework called a cell [1]. Cellular structures have a wide range of applications, including in biomedicine [2,3,4], aerospace [5,6,7], civil [8,9,10], and automotive industries [11,12,13], to name but a few [14,15,16,17,18,19,20,21,22,23]. While traditional cellular structures consist of uniform patterns (i.e., uniform cell densities), they can be constructed with spatially varying shapes, sizes, and cell orientations to achieve optimized performance in regard to various different types of applications and to achieve the desired combination of properties, such as high strength [24], lightweight [25], effective heat dissipation [26], etc. [27,28,29,30,31,32,33,34]. The constitutive response and optimal design of cellular structures with uniform and spatially varying patterns have been investigated by numerous researchers in the past [35,36,37,38,39,40,41,42,43,44]. Spatially graded cellular structures can be found in nature with different scales, ranging from nanometers to meters. Examples are diatoms [45], butterfly wings [46], grass stems [47], dentin [48], sea urchin spines [49], and bone [50]. Such spatially graded natural patterns have inspired many researchers during the design and building of optimal artificial components, by studying the structural response at different levels of hierarchy [51,52,53,54,55,56,57,58,59]. For example, an artificial bone can be constructed by mimicking the hierarchical porous structure of a bone at different scale lengths and can be further optimized in regard to different loading conditions, using various optimization techniques. Recent advances in additive manufacturing (AM) techniques have enabled researchers to precisely build cellular structures with different dimensions using different shapes, orientations, and cell sizes, ranging from microns to meters [60,61,62,63,64,65,66,67,68,69], allowing the micromechanical constitutive behavior of micro-sized cells to be tailored in regard to the macroscopic structural response. However, it is well known that the manufacturing process (e.g., the printing direction) can significantly affect the mechanical response of the component [70,71,72,73,74,75,76]. As a result, additively manufactured spatially graded microcellular structures may exhibit highly anisotropic behavior, as a result of both the geometrical configurations (cell pattern) and the material properties (printing direction).

The analysis of cellular structures is indeed a significant challenge, due to the inherent intricacy of such materials. One way to avoid this complex task is to utilize homogenization methods, according to which the original non-continuous structure is equalized with a homogeneous analogous medium, where both structures exhibit the same macroscopic material properties. In regard to this technique, the heterogeneous structure is divided into small parts, known as representative volume elements (RVEs), and analyzed to determine their material properties. Then, the obtained properties are integrated and then averaged to form a continuous medium, with the same overall material behavior as the original non-homogenous one. In other words, homogenization is a bridge to cover the gap between the microscale behavior of cellular materials and the macroscale requirements of engineering applications [77].

Several methods have been proposed and utilized for numerical homogenization. These methods include, but are not limited to, Bloch’s theorem and the Cauchy–Born hypothesis [78], where the former theorem is used to describe the wave behavior in periodic structures and the latter associates the deformation of a crystal lattice with the macroscopic strain in the material, and, together, they determine the relationship between the microscale and macroscale performance of the medium. Some popular approaches include micropolar theory [79,80,81], which extends classical continuum mechanics to account for microstructural effects by considering the microscopic rotation of the particles within the material; the strain energy equivalence method [82,83], which equates the strain energy in the microstructure of the material with that of an equivalent homogeneous medium; the beam theory approach [37,84,85], which utilizes the principles of beam mechanics by simplifying the structure into a sequence of beams and, finally, computes the overall properties based on the properties and arrangement of these beams; the multi-scale homogenization method [86], which integrates the material behavior information obtained from across the microscale to the macroscale in order to make a more accurate approximation of the overall properties of the medium; the machine learning approach [87], which uses algorithms to foresee the homogenized properties of a material by using microstructural data; and the asymptotic homogenization (AH) approach [88], which is a mathematical technique that uses asymptotic expansions to estimate the macroscale behavior of the material. If the homogenization equation is discretized and solved using finite element analysis (FEA) or other numerical methods, it is commonly called numerical homogenization. The advantages of this approach are abundant. For example, this procedure can be conducted on a wide range of materials and various microstructures with anisotropy or different complexities (whether they are periodic or non-periodic) and it can be easily customized or integrated with other methods. Andreassen and Andreasen [89] used the theory of homogenization and presented a MATLAB R2023a code to calculate the macroscopic elasticity tensor of two- or multi-material systems made of *isotropic* materials (where one of the materials could be void) with *uniform* patterns of voids. Also, they described and extended their code for the homogenization of fluid permeability, thermal expansion, and conductivity. Later, Dong et al. [90] used this code and expanded it to achieve a homogenized constitutive matrix of 3D cellular materials or multi-material composites.

In this work, several MATLAB codes are developed to obtain the homogenized material properties of microcellular materials with *functionally graded void patterns*, made from *both isotropic and orthotropic materials*. To this end, first, a separate code is developed to build the desired periodic functionally varying cellular structure with different void shapes (circular, hexagonal, and rectangular/square). Then, numerical homogenization is adopted to compute homogenized elasticity tensors assigned to the centroid of each unit cell. If the material in the solid phase is isotropic, the approach presented in [89] is taken (herein known as the “reference elasticity tensor”). However, the MATLAB implementation in [89] cannot consider material anisotropy. To address this deficiency, a new homogenization code was developed herein, which includes a parameter called the *printing angle*. The overall microcellular structure is achieved by stacking unit cells, where each unit cell is treated as a point. A fit function is then assigned to create a continuous surface from the discrete material points (centroids of unit cells). This approach is advantageous because it allows for the prediction of the elasticity tensor at other points in the homogenized material domain. By comparing the reference elasticity tensor and the predicted one, the accuracy of the current computations is evaluated. If the material in the solid phase is isotropic, the outputs are the reference and the predicted elasticity tensors (at any desired points), the fit function that maps the entire medium and its coefficients for each elasticity tensor component, the overall average element-wise percentage error, and the plots of the microcellular structure and the corresponding relative density. If the material in the solid phase is orthotropic, the outputs are the same but for both global coordinates and axes of orthotropy and the plots of material properties of the entire domain. Note that the developed codes are well capable of considering macroscopic anisotropy, whether it comes from the orthotropic behavior of the solid phase or the geometry of the unit cell. Also, this methodology is only suitable for periodic void patterns since non-periodic patterns lack a repeating unit cell that can serve as a representative volume element (RVE). Without a well-defined RVE, it becomes challenging to homogenize the material properties accurately, as the microstructural variations cannot be captured by a single representative sample. If non-periodic void patterns are in mind, alternative modeling approaches such as direct numerical simulations or stochastic homogenization methods can be utilized, which are more complex and often increase the computational cost. Furthermore, it is worth mentioning that the presented MATLAB implementation can be used in the homogenization of composite materials with more than one orthotropic phase. At the end, some examples are solved, and the accuracy of the numerical implementation and its capabilities are addressed. Future work could focus on incorporating advanced materials (such as nanoparticles and multifunctional composites [91,92]) or materials that exhibit non-linear behavior, into homogenization frameworks.

## 2. Theoretical Framework and MATLAB Implementation

By assuming that the size of the unit cell is significantly smaller than the entire cellular structure (i.e., microcellular materials) and the bonding between different length scales/materials is perfect, the theory of elasticity describes the macroscopic stiffness tensor $C_{ijkl}^{H}$ using the following equation:

(1) $$C_{ijkl}^{H}=\frac{1}{\left|V\right|}\int_{V}C_{pqrs}\left(\varepsilon_{pq}^{0(ij)}-\varepsilon_{pq}^{\left(ij\right)}\right)\left(\varepsilon_{rs}^{0(kl)}-\varepsilon_{rs}^{\left(kl\right)}\right)dV,$$

where $\left|V\right|$, $C_{pqrs}$ and $\varepsilon_{pq}^{0(ij)}$ are the volume of the unit cell, the locally varying stiffness tensor, and the prescribed macroscopic strain fields, respectively [89,93]. Moreover, $\varepsilon_{pq}^{\left(ij\right)}$ is the locally varying strain fields, which are defined as follows:

(2) $$\varepsilon_{pq}^{\left(ij\right)}=\varepsilon_{pq}\left(\chi^{ij}\right)=\frac{1}{2}(\chi_{p,q}^{ij}+\chi_{q,p}^{ij}).$$

The displacement fields ($\chi^{kl}$) can be found by solving the following equation:

(3) $$\int_{V}C_{ijpq}\varepsilon_{ij}\left(\upsilon \right)\varepsilon_{pq}\left(\chi^{kl}\right)dV=\int_{V}C_{ijpq}\varepsilon_{ij}\left(\upsilon \right)\varepsilon_{pq}^{0(kl)}dV\;\;\;\;\;\;\;\;\;\;\;\;\;\;\;\;\;\;\;\;\;\;\;\;\;\forall \upsilon \epsilon V,$$

where υ is the virtual displacement field. Generally, Equation (3) is solved numerically by discretizing the cell domain. To this end, both the left-hand side (i.e., the stiffness matrix) and the right-hand side (i.e., the mechanical force vectors due to the macroscopic unit strains $\varepsilon^{0}$) of this equation need to be discretized. To calculate displacement fields in Equation (3) by using FE methods, we proceed as follows:

(4) Kχ=F,

where ***K*** is the stiffness matrix and F is the mechanical force vector due to the corresponding macroscopic unit strains, where, herein, the strains are chosen to be:

(5) $$\varepsilon_{1}^{0}={\left(1,0,0\right)}^{T},\varepsilon_{2}^{0}={\left(0,1,0\right)}^{T},\;\varepsilon_{3}^{0}={\left(0,0,1\right)}^{T}.$$

Equation (5) physically means that in the first case, the unit strain is applied along the x-axis; in the second case, the unit strain is applied along the y-axis; and the last case corresponds to a pure shear strain. Consequently, the force vectors can be calculated by:

(6) $$F=\sum_{e=1}^{N}\int_{V_{e}}B_{e}^{T}C_{e}\varepsilon^{0}{dV}_{e},$$

where *N*, $B_{e}$, $C_{e}$, and $V_{e}$ are the total number of elements in a unit cell, the element strain displacement matrix, the element stiffness matrix, and the volume of the element, respectively.

Regarding the left-hand side of Equation (4), for the stiffness matrix, we have:

(7) $$K=\sum_{e=1}^{N}\int_{V_{e}}B_{e}^{T}C_{e}B_{e}{dV}_{e},$$

where the element stiffness matrix ($C_{e}$) for an isotropic material depends on Lame’s first and second parameters and they can be, respectively, found using Equations (8) and (9) as follows:

(8) $$\lambda =\frac{\nu E}{\left(1+\nu )(1-2\nu \right)},$$

(9) $$\mu =\frac{E}{2(1+\nu )},$$

where *E* is the Young’s modulus and *ν* is the Poisson’s ratio. If the plane stress conditions are in mind, Lame’s first parameter can be modified and used as:

(10) $$\hat{\lambda }=\frac{2\mu \lambda }{\lambda +2\mu }.$$

**Figure 1:**
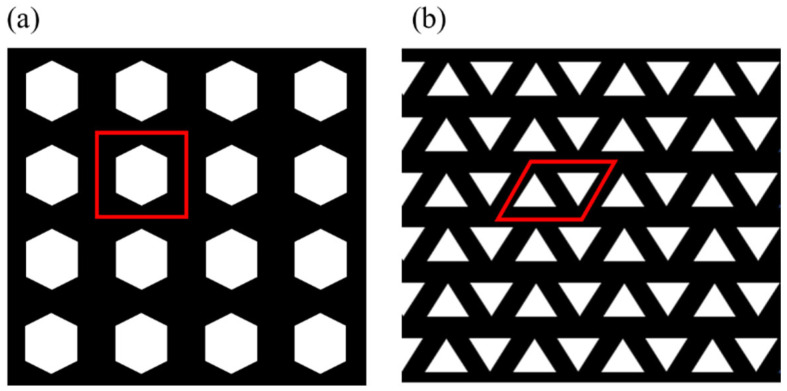
Examples of 2D periodic cellular patterns with parallelogram-shaped unit cells containing (**a**) hexagonal and (**b**) triangular voids.

The mentioned procedure is often referred to as numerical homogenization and has been widely utilized by various researchers in the past [89,90,91,92,93]. Figure 1 shows two examples of different 2D periodic cellular patterns, where each cell can be described by the parallelogram-shaped unit cells. Note that the shape of the unit cell is greatly influenced by the overall structure of the cellular domain. The unit cell should be designed to best capture the periodic repetition of the voids (see Figure 1)

**Figure 2:**
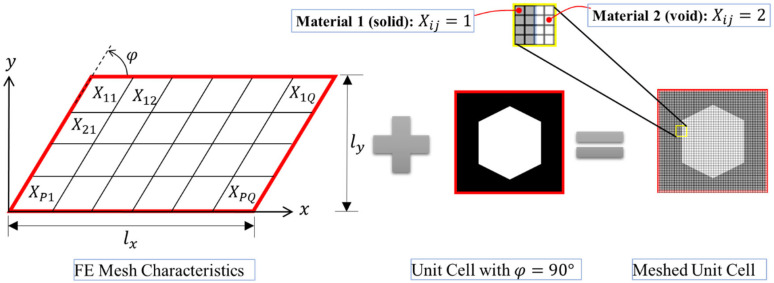
The structure of the FE mesh and its application on a unit cell consisting of two material phases.

In this approach, the unit cell itself will be discretized and then solved by finite element (FE) methods. Figure 2 illustrates the structure of the FE mesh and its corresponding geometrical constraints, together with the actual meshed unit cell consisting of two material phases. In this case, an indicator matrix *X* denotes whether the element contains material one ($X_{e}=1$) or material two ($X_{e}=2$). As can be seen in this figure, the unit cell can conveniently be characterized by three geometrical parameters of width (*l_x_*), height (*l_y_*), and the angle (*φ*) between the x-axis and the left wall of the unit cell. To avoid overly distorted elements, a range of 45°≤ *φ* ≤ 135° is usually recommended [89].

As discussed earlier, Andreassen and Andreassen [89] have developed a MATLAB code for the homogenization of composite materials, assuming that the constituent phases are *isotropic*. A comprehensive explanation and possible extensions of the code are thoroughly addressed in [89], so further explanations on this matter are avoided herein for the sake of brevity. However, for the reader’s convenience, the code is reported in Appendix A by the name of *homogenize*. The inputs for this code are the dimensions of the unit cell (i.e., *l_x_* and *l_y_*); Lame’s first and second parameters for the materials (in this work, the focus is on the homogenization of functionally graded periodic cellular materials so one material is associated with the solid part of the structure and the other one is void); the angle formed by the left wall of the unit cell and the x-axis (i.e., *φ*); and the indicator matrix, *X*, which has two purposes. Firstly, it in indicates what material is within the unit cell, and, secondly, the size of this matrix characterizes how fine the discretization is (see Figure 2). Thus, the function can be called (note that “flag” is just a debugging parameter):



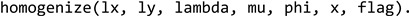



In the current work, two new sets of codes (functions) are developed to model an isotropic cellular structure and to appropriately utilize the *homogenize* code to obtain the corresponding elasticity tensor of the homogenized material domain at any point. The first developed code is called *Cellular_Solid* (see Appendix B), which is responsible for making the unit cell with the desired void and requires three inputs, including the size of the mesh grid (i.e., the size of indicator matrix), the shape of the void, and an “Argument”, which specifies the size of the void. In the present work, the void shapes that this code supports are circles, hexagons, squares, and rectangles. The variable (or the argument) that determines the size of the circular or the hexagonal void is the radius and half of the hexagon’s diagonal, respectively. Changing these parameters will alter the size of the voids, much like using a magnifying tool. However, for the rectangle, two variables of width and height are required, and if the two are equal, a square is expected. The second code generated herein is called *Homogenize_test* (see Appendix C), and it properly and efficiently uses the other two codes to construct an isotropic periodic cellular structure to collect elasticity tensor data from all discrete points of the homogenized structure to create a continuous function that can estimate the tensor components at any point within the homogenized domain. The first part of this code is designated to initialization and collects four user inputs, including unit cell’s matrix size, the unit cell’s void shape, and the dimensions of the overall structure. Then, it establishes the grid parameters to define the range for x and y coordinates according to the input dimensions of the structure. Each x and y coordinate pair represents the centroid of a unit cell and can be used as a variable for the argument which identifies the size of the void. This is beneficial, because by assigning a function to the corresponding argument, different void sizes can be achieved by moving from one material point to another. Then, the code saves two main data for each point. The first one is the reference elasticity tensor for the corresponding unit cell obtained from the *homogenize* function. The second one is relative density, obtained by analyzing the material distribution within each unit cell by computing the sum of elements in the matrix *X* that contains material one (in this case, solid material) divided by the total number of elements. Afterwards, the final structure and the corresponding relative density are plotted. Then, both the relative density and the elasticity tensors are curve fitted by using a predefined function in MATLAB (in this case, “poly55”). Also, the overall average element-wise percentage error for the obtained tensor function is calculated by first obtaining the percentage error for each tensor element by using the following equation:

(11) $$Percentage\;Error=\left|\frac{Reference\;value-Predicted\;value}{Reference\;value+\epsilon }\right|\times 100,$$

where ϵ is a small constant added to avoid division by zero. Then, these individual percentage errors are summed across all tensor elements for all unit cells and, finally, divided by the total number of material points. It is worth noting that in the current work, poly55 is used for its simplicity, computational efficiency, acceptable fitting accuracy, and convenience in polynomial surface fitting. Obviously, any other fitting function can be used herein, including user-developed ones. Note that for complex geometries, it is recommended to use other methods like spline fitting instead of using higher-degree polynomials, since it might result in computational overhead or overfitting. Moreover, if desired, the reference and the fitted elasticity tensors at any specified point can be displayed as an output by hard coding the coordinates of that point.

**Figure 3:**
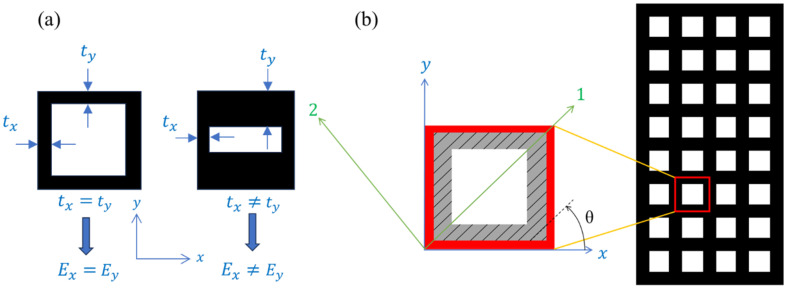
(**a**) An example of geometrically anisotropic cell, (**b**) An orthotropic periodic cellular structure featuring square voids, printing angle θ, with global x-y and principal 1–2 coordinate systems.

Recently developed, complex, and ultra-precise additive manufacturing methods have enabled the creation and utilization of unique microcellular structures with functionally graded patterns and tailored mechanical properties. The homogenization of such topologically complex components is a crucial task for analyzing and predicting their behavior under various loading conditions. Due to the nature of this manufacturing process which is performed layer by layer at a specified angle, the final structure often exhibits significant orthotropy in the printing direction [93]. Despite the valuable contributions of Andreassen and Andreassen [89] and although their method is capable of considering anisotropy induced by cell topology, it fails to consider orthotropic material phases in the homogenization approach. Therefore, their approach may not be suitable for additively manufactured microcellular structures, which are the focus of this work. Figure 3a,b show two examples of anisotropies caused by the topology of the cell and the printing direction, respectively.

Up to this point, the focus of the current method has been on the homogenization of isotropic functionally graded periodic cellular structures, for which two new sets of codes have already been provided. However, the core novelty and innovation of the present work lie in developing a new code capable of considering material orthotropy in the homogenization process as well as spatially varying cellular patterns. The inputs of this new code, which is called *homogenize_ortho* (see Appendix D), are the same as the ones that are used in the *homogenize* function, but since the material orthotropy is considered, Lame’s first and second parameters are omitted. Instead, five new inputs of $E_{1}$ (Young’s modulus in first principal direction), $E_{2}$ (Young’s modulus in second principal direction), $G_{12}$ (intralaminar shear modulus), $\nu_{12}$ (the Poisson’s ratio in the 2-direction due to load being applied in the 1-direction), and θ (the printing or the orthotropy angle) are added, so the final form of the line is shown below:



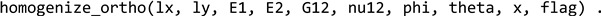



After receiving the required inputs, the code transforms the material properties from principal directions to a global x-y coordinate system by using the well-known transformation equations for orthotropic materials as follows:

(12) $$E_{x}={\left(\frac{{cos}^{4}(\theta )}{E_{1}}+\left(\frac{1}{G_{12}}-\frac{2\nu_{12}}{E_{1}}\right){sin}^{2}\left(\theta \right){cos}^{2}\left(\theta \right)+\frac{{sin}^{4}(\theta )}{E_{2}}\right)}^{-1},$$

(13) $$E_{y}={\left(\frac{{sin}^{4}(\theta )}{E_{1}}+\left(\frac{1}{G_{12}}-\frac{2\nu_{12}}{E_{1}}\right){sin}^{2}\left(\theta \right){cos}^{2}\left(\theta \right)+\frac{{cos}^{4}(\theta )}{E_{2}}\right)}^{-1},$$

(14) $$G_{xy}={\left(\frac{1}{G_{12}}\left({sin}^{4}\left(\theta \right)+{cos}^{4}\left(\theta \right)\right)+4\left(\frac{1}{E_{1}}+\frac{1}{E_{2}}+\frac{2\nu_{12}}{E_{1}}-\frac{1}{{2G}_{12}}\right){sin}^{2}\left(\theta \right){cos}^{2}\left(\theta \right)\right)}^{-1},$$

(15) $$\nu_{xy}=E_{x}\left(\frac{\nu_{12}}{E_{1}}-\frac{1}{4}\left(\frac{1}{E_{1}}+\frac{2\nu_{12}}{E_{2}}\frac{1}{E_{2}}-\frac{1}{G_{12}}\right){sin}^{2}\left(2\theta \right)\right).$$

For orthotropic materials, the stiffness matrix *C* is commonly represented as:

(16) $$C=\left[\begin{array}{ccc} E_{x} & \nu_{xy}E_{y} & 0\\ \nu_{yx}E_{x} & E_{y} & 0\\ 0 & 0 & G_{xy} \end{array}\right],$$

The transformed material properties are assigned to each element. After that, element-level stiffness matrices (keC) and force vectors (feC) are calculated based on the geometry of the unit cell. Once the boundary conditions and the global stiffness matrix and load vector are defined, the code finds the displacement field (chi) inside of the unit cell under three typical load cases of axial strain in the x-direction (epsilon0_11 = (1, 0, 0)), axial strain in the y-direction (epsilon0_22 = (0, 1, 0)), and shear strain (epsilon0_12 = (0, 0, 1)). Finally, the homogenized elasticity tensor (CH) is calculated by integrating the stress and strain fields over the volume of the unit cell, which provides a macroscopic view of how the material behaves as a continuous medium despite its microscopic heterogeneities.

To effectively use the *homogenize_ortho* function, an innovative code, named *Homogenize_test_ortho_principal,* has been developed (see Appendix E). Like before, this code makes a connection between the *Cellular_Solid* function and creates the unit cells, then stacks these cells to make the overall cellular structure. Each unit cell is represented by a point in the structure, and the corresponding elasticity tensor and relative density are assigned to that point. However, an important issue herein is that the obtained elasticity tensor from the *homogenize_ortho* function is in the global coordinate system and not in the principal directions. To solve this issue, creatively, Equations (12)–(15) are solved backwards to obtain $E_{1}$, $E_{2}$, $G_{12}$, and $\nu_{12}$ as follows (note that eqn1, eqn2, eqn3, and eqn4 are the same as $E_{x}$, $E_{y}$, $G_{xy}$, and $\nu_{xy}$, respectively, as shown in Equations (12)–(15)):



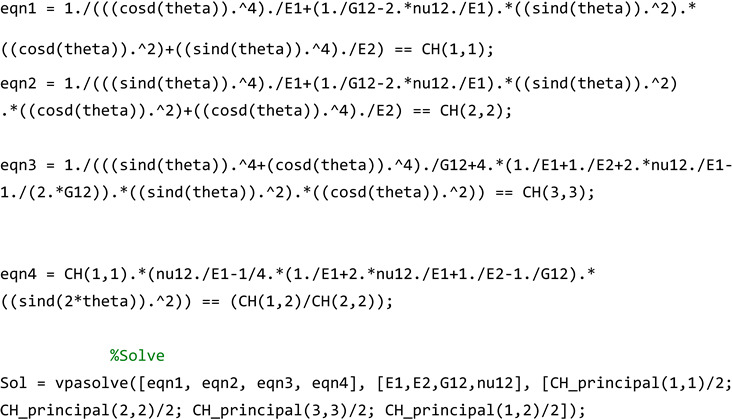



Note that the initial guesses need to be hard coded at the beginning of the code in the following lines (fill the blank):



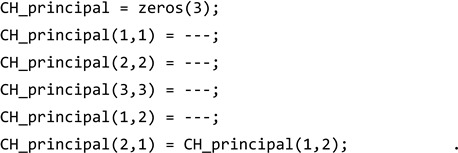



**Figure 4:**
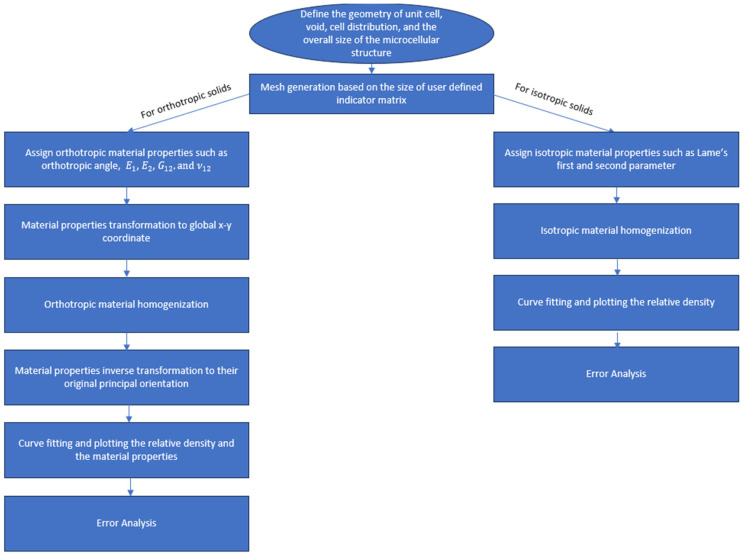
The flowchart of the homogenization process for orthotropic and isotropic solid phase in periodic functionally graded microcellular materials.

Now that the material properties in the principal directions are obtained, they are substituted into Equation (16) to obtain the reference elasticity tensor at each material point. The remainder of the code performs the same operations discussed earlier for the *Homogenize_test* function with the added features of plotting $E_{1}$, $E_{2}$, $G_{12}$, $\nu_{12}$. Moreover, the coefficients of the polynomial fitting functions for elasticity tensors and relative density distributions are documented in CSV files. Also, note that, in this case, the tensor functions for both global x-y and principal 1–2 coordinate systems will be displayed. Figure 4 depicts a simple flowchart of the explained procedure.

## 3. Results and Discussion

In this section, several examples of homogenizing periodic functionally graded cellular structures, considering both orthotropic and isotropic cases, are presented. For the isotropic ones, six different cases are considered. Note that before running the code, the inputs for the *homogenize* function need to be hard coded by making changes in the following line:



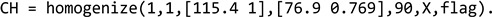



As mentioned, the first two inputs are *l_x_* and *l_y_* (the dimensions of the unit cell in x and y directions), which, in this example, are both equal to unity. The third input is a one-by-two matrix, where the first entry is Lame’s first parameter for the first material (in this case, the solid phase) and the second entry is Lame’s first parameter for the second material (which is void in this work). Note that, according to [89], when dealing with the void, it is recommended to use one-hundredth of the value used for the solid material. The next input is Lame’s second parameter, and it follows the same rule as Lame’s first parameter. Note that, herein, a hypothetical material with a Young’s modulus of 200 GPa and a Poisson’s ratio of 0.3 is considered for the solid phase. Note that by substituting these values in Equations (8) and (9), Lame’s first and second parameters for the solid material are computed. The fifth input is the angle between the horizontal and the inclined wall of the unit cell, φ, in degrees, which, in this example, is equal to 90°. This means that by considering the given *l_x_* and *l_y_*, the unit cell is a one-by-one square. Finally, X is the size of the indicator matrix used for the discretization of the unit cell, and its size will be established once the code is run, so no changes are required here. To summarize it, the hard coded inputs are *l_x_* = 1, *l_y_* = 1, Lame’s first parameter for the solid is 115.4, Lame’s first parameter for the void is 1, Lame’s second parameter for the solid is 76.9, Lame’s second parameter for the void is 0.769, *φ* = 90 (meaning the unit cell is square given that its width and height are equal to unity), and, finally, the size of the indicator matrix, which does not need to be hard coded since it will be checked once the code is run.

Once these inputs are hard coded, the *Homogenize_test* can be run. When the code is run, it asks for four inputs of UC (unit cell) matrix size, UC’s void shape, the structure’s width, and the structure’s height. Here, a unit cell matrix size of 50 is utilized. This means that the cell will be discretized with a 50-by-50 mesh. Regarding the void shape, three cases of circle, rectangle, and hexagon have been defined, and to choose any of the aforementioned geometries, the user can simply type the name of the shape. Note that the desired function needs to be hard coded in the corresponding void size argument. The input for both the width and height of the structure is chosen to be 20. This means that the entire cellular material domain consists of 400 unit cells (and, therefore, material points) stacked together.

**Figure 5:**
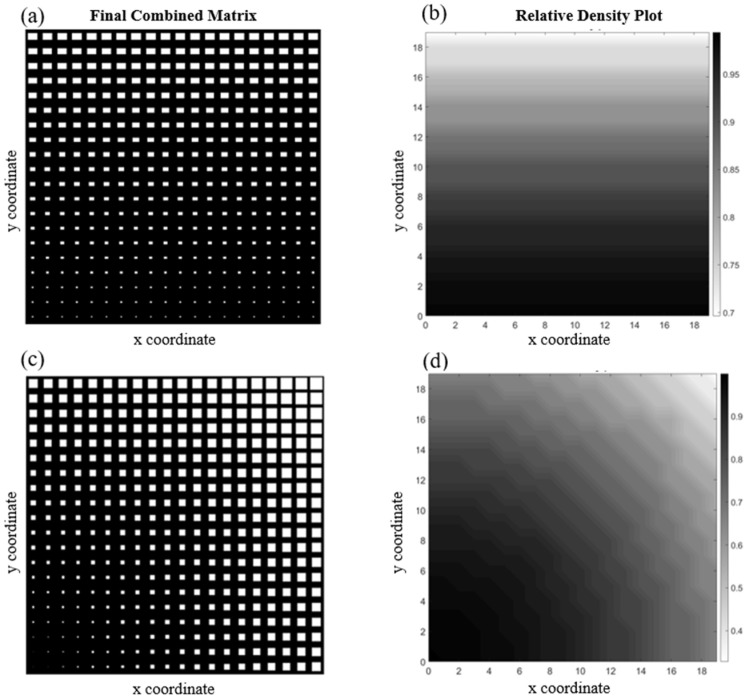
Two functionally graded cellular structures with (**a**) rectangular voids that increase in size in the y-direction and (**b**) its relative density plot together with (**c**) a structure with diagonally increasing square voids and (**d**) the corresponding relative density plot.

Figure 5 shows two functionally graded cellular structures (Figure 5a,c) with their corresponding relative density plot (Figure 5b,d). The first structure is a cellular medium with varying rectangular-shaped voids in the y-direction, while the voids in the other one have the shape of a square, and their sizes change in the diagonal direction. Both structures have simple patterns, resulting in smooth changes in material properties from one point to another. Consequently, applying a fitting function capable of giving a good approximation for the actual data (i.e., the reference elasticity tensor obtained from the *homogenize* function) will not be difficult. This is confirmed by the relatively small amounts obtained as overall average element-wise percentage error, which are 1.09% and 1.91% for the cases illustrated in Figure 5a,c, respectively.

**Figure 6:**
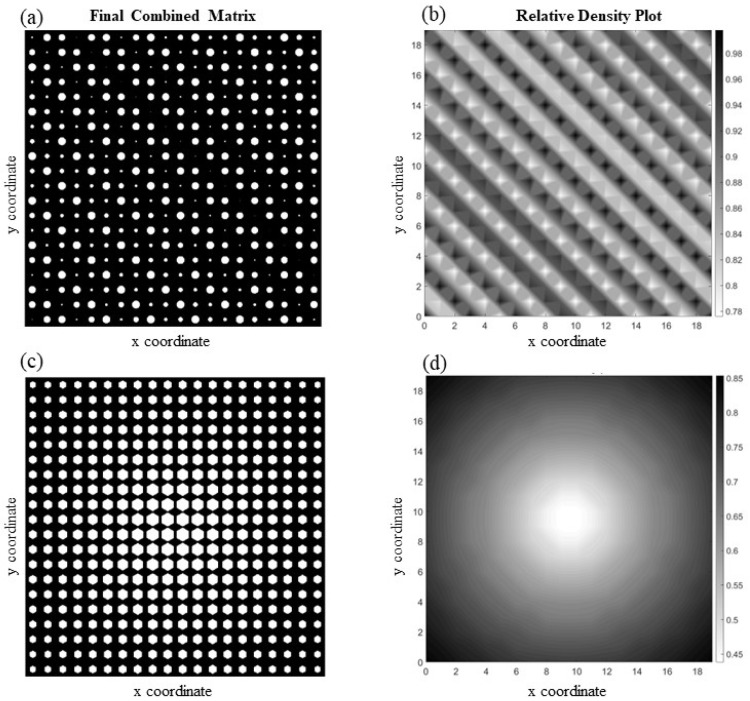
Two examples of functionally graded cellular structures with isotropic material phases: (**a**,**b**) a diverse circular void pattern and its corresponding relative density plot; (**c**,**d**) hexagonal voids and the corresponding relative density plot.

There is an intriguing phenomenon hidden in the structure shown in Figure 5a. Even though the material of the solid phase is isotropic, the structure exhibits anisotropy at the unit cell level due to the geometry of the voids. Note that, unlike Figure 5c, the unit cells are not symmetric in both the x and y directions (cf. Figure 3a). Therefore, the unit cell returns different values of elastic modulus along the x and y directions, computed herein as $E_{x}=$161.53 GPa and $E_{y}=$128.92 GPa. Note that elongated voids aligned in the x direction make the material stiffer along that direction. A high or random discrepancy in the void sizes across different unit cells leads to significant variations in material properties, making it mathematically challenging to develop a function that can accurately predict these properties. For instance, Figure 6a depicts a structure with circular voids and a highly diverse void pattern. As can be seen in this example, the size of the voids and their position change dramatically, both pattern-wise and size-wise, so assigning a fit function will be challenging. For this reason, it is not surprising to see that the overall average element-wise percentage error in this case is 13.95%. Meanwhile, Figure 6c shows a structure with hexagonal void shapes, where slower changes in void sizes occur as we move from the center to the edges of the medium. In addition to that, the stochasticity in the pattern is much less than that of 5a, resulting in an overall average element-wise percentage error of 1.51%. The relative density for both structures is depicted in Figure 6b,d. 

**Figure 7:**
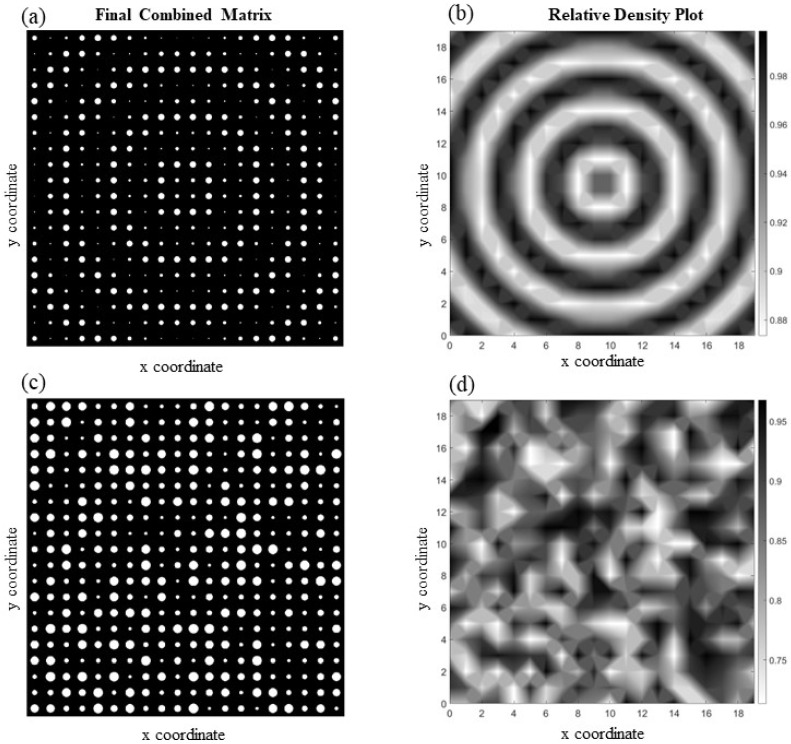
Examples of complex periodic microcellular structures: (**a**,**b**) illustrate a medium with circular voids that resembles a waive alongside its corresponding relative density plot; (**c**,**d**) present a structure containing random circular voids and its associated relative density plot.

Two highly diverse structures in terms of void size and pattern are illustrated in Figure 7a,c. Figure 7a depicts a cellular structure with a pattern of circular voids that change diagonally, resembling the rippling effect of water waves as one droplet of water merges into another, and the structure in Figure 7c has random size voids in each unit cell. The complexity in both structures is noticeable in the density plots illustrated in Figure 7b,d. Due to the complex characteristics of the voids in these structures, it is not surprising to see that the overall average element-wise percentage error for the first structure is 7.44% and for the second one is 13.73%, which is high.

**Figure 8:**
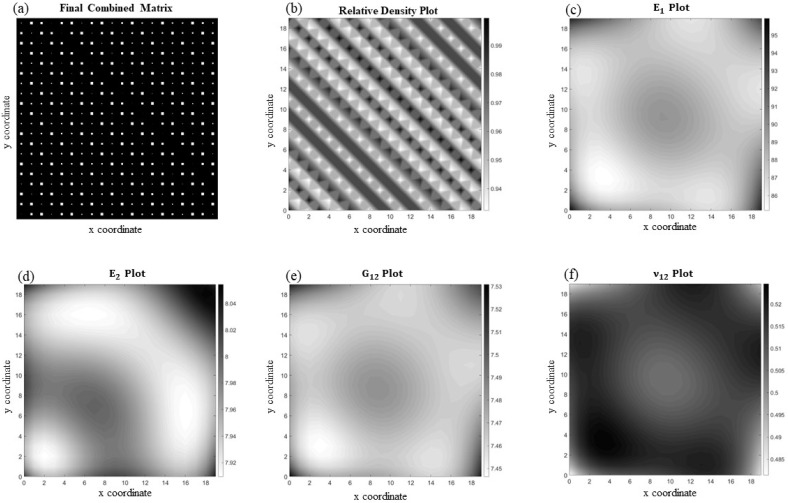
(**a**) A periodic functionally graded microcellular structure with orthotropy angle of 30° and its associated homogenized material properties including (**b**) relative density, (**c**) $E_{1}$, (**d**) $E_{2}$, (**e**) $G_{12}$, and (**f**) $\nu_{12}$.

**Figure 9:**
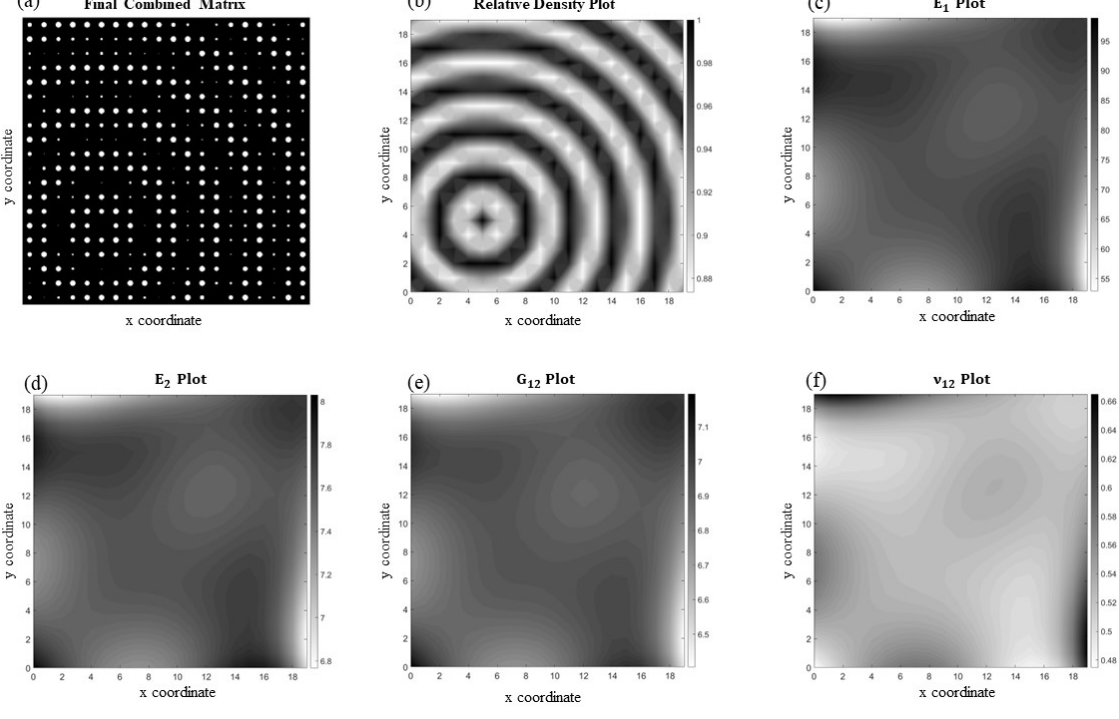
(**a**) A complex periodic microcellular structure with orthotropy angle of 60° and its corresponding homogenized material properties, including (**b**) relative density, (**c**) $E_{1}$, (**d**) $E_{2}$, (**e**) $G_{12}$, and (**f**) $\nu_{12}$.

For the orthotropic functionally graded periodic cellular materials, two examples are shown in Figure 8 and Figure 9. For this part, the appropriate homogenization code, *Homogenize_test_ortho_principal*, needs to be used, and note again that, before running the code, the inputs must be hard coded as:



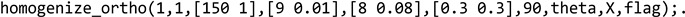



Like the isotropic example, the first two inputs are the unit cell dimensions (both are set to one). The third input is a matrix where the first entry is $E_{1}$ for material one (in this case, 150 GPa) and the second entry is $E_{1}$ for material two. The fourth through sixth entries are matrices associated with $E_{2}$, $G_{12}$, and $\nu_{12}$. Note that, once more, the mentioned rule is applied here, in which for the void, one-hundredth of the value used for the solid material is used. The next entries are *φ* = 90°, the printing angle (θ), and finally the size of the indicator matrix. The last two values, the printing angle and matrix size, will be prompted for input from the user once the code is run, so no prior changes are required.

Figure 8 shows an orthotropic periodic microcellular structure with square voids and a printing angle of 30° (Figure 8a) along with its corresponding relative density, $E_{1}$, $E_{2}$, $G_{12}$, and $\nu_{12}$ plots (Figure 8b–f, respectively). Even though the patten seems to be complicated, the variation in the void size is not significant, which makes it relatively easy to fit a function capable of predicting the datapoints with acceptable precision. This is verified by knowing that the overall average element-wise percentage error for this case is 3.44%.

Figure 9 illustrates an orthotropic periodic functionally varying microcellular structure but with a more complex pattern (Figure 9a), which resembles a ripple emanating from the lower left of the medium. The orthotropy angle (i.e., printing orientation angle) is 60°, and the unit cells contain circular voids, with sizes that change more dramatically compared to the previous example. This dissimilarity leads to considerable changes in the material properties at each unit cell and consequently makes it difficult to fit a function capable of accurately capturing these variations. This results in an overall average element-wise percentage error of 6.80% for this case. The corresponding homogenized material properties are depicted in Figure 9b–f.

## 4. Conclusions

Recent developments in additive manufacturing have not only broadened the horizon for creating structures with intricate features (like microcellular solids) but have also accentuated the necessity of understanding and analyzing them under various loading conditions. Homogenization is a powerful computational tool for the simplification of materials with complex discrete properties into an equivalent continuum medium. This technique is vital in various fields, particularly in structural and fracture analysis, where simulating crack nucleation and propagation in complex (i.e., heterogeneous) materials is not only complicated but also computationally expensive [94,95,96,97,98,99,100]. In the current method, for the first time, homogenization is utilized to obtain the macroscopic elasticity tensor of functionally graded periodic cellular materials, considering both isotropic and orthotropic constituents, from the equivalent homogeneous counterparts. To this end, several MATLAB implementations are developed and presented for future users. Regarding structures with isotropic solids, two new sets of codes are developed. The first one, named *Cellular_Solid*, defines both the geometry of the unit cell and the void inside of it, while the other one, named *Homogenize_test*, has several duties. Firstly, it retrieves the unit cell from *Cellular_Solid* and then effectively utilizes a homogenization code already reported in the literature (herein by the name of *homogenize*) to obtain the homogenized unit cell’s elasticity tensor. Each unit cell is then stacked up, and the final structure is generated and plotted along with its corresponding relative density. This code treats the unit cells as material points and assigns the homogenized elasticity tensor to their centroids. Finally, using a predefined MATLAB fit function, namely “poly55” in the current work, this code maps the medium and predicts the elasticity tensor at any given material point. 

The primary innovation of this work lies in analyzing spatially varying cellular materials while considering the effects of material orthotropy in the solid phase, to account for the orthotropic nature of these materials fabricated via additive manufacturing. For this case, another two sets of codes are developed. The first code is a novel homogenization code, which is capable of considering material orthotropy. Finally, the last code, which is called *Homogenize_test_ortho_principal,* makes a connection between other codes and not only plots the structure and its relative density but also illustrates $E_{1}$, $E_{2}$, $G_{12}$, and $\nu_{12}$ for the equivalent homogeneous medium. In both the isotropic and orthotropic cases, the overall average element-wise percentage error is reported too. This work showed that by using the MATLAB implementation provided herein, the homogenized elasticity tensors in both isotropic and orthotropic (whether it is due to the material or the asymmetrical geometry of the void) functionally graded periodic cellular materials can be obtained accurately, conveniently, and with low computational costs. One of the advantages of the current method is that each entry of the final elasticity tensor is an equation that maps the entire domain. Having an output like that is beneficial because it can be easily used in advanced fracture models such as XFEM or peridynamics. Also, it was revealed that the total accuracy of the predicted macroscopic elasticity tensor highly depends on the complexity of the void patterns and how severe the void sizes change from cell to cell. It was shown that the more complex they become, the harder it is to fit a function that can accurately represent each material point. Thus, the fitting function plays an important role in the overall accuracy; as the stochasticity in the structure increases, a more sophisticated fitting function is required. For instance, utilizing a fit function based on Furrier series might be a prudent choice since it is especially useful for modeling periodic data, due to its ability to represent any periodic function by decomposing it into a sum of sine and cosine terms. Moreover, it was revealed that as the relative density decreases (i.e., porosity increases), the material’s stiffness and strength normally decrease, and the geometry-induced anisotropy becomes more pronounced due to the large void size.

## Data Availability

The data and MATLAB codes supporting the reported results are provided in the Appendix of this article. Additional data or materials can be requested from the corresponding author.
